# Editorial: Advances in imaging of pediatric heart diseases

**DOI:** 10.3389/fped.2023.1305566

**Published:** 2023-10-12

**Authors:** Liqun Sun, Harvey Ho, Xiaojuan Ji

**Affiliations:** ^1^ Division of Cardiology, Hospital for Sick Children, University of Toronto, Toronto, ON, Canada; ^2^ Auckland Bioengineering Institute, University of Auckland Auckland, Auckland, New Zealand; ^3^ Department of Ultrasound, Chongqing General Hospital, Chongqing, China

**Keywords:** pediatric heart diseases, echocardiography, computed tomography, cardiovascular magnetic resonance, fetal echocardiography, fetal cardiovascular magnetic resonance

**Editorial on the Research Topic** Advances in imaging of pediatric heart diseases

## 1. Advanced imaging in fetal heart diseases

Over the preceding decade, multiple guidelines on fetal echocardiography have been disseminated for the comprehensive evaluation of the fetal cardiac structure. The updated fetal cardiac screening guideline established by the International Society of Ultrasound in Obstetrics and Gynecology (ISUOG) introduced a framework involving examining the fetal heart (1). Notably, Behera et al. (2) elucidated that enhancing fetal heart imaging quality is pivotal in mitigating avoidable cognitive errors, emphasizing the significance of quality improvement initiatives. Furthermore, Moon-Grady et al. (3) expounded upon the research design of the fetal echocardiographic Z Score pilot project, shedding light on the influence of gestational age and variable types on the reproducibility of measurements, both within and across different investigators. In 2023, Szabo and colleagues provided an overview of the primary techniques employed in fetal cardiovascular magnetic resonance imaging (CMR). They also delved into the intricacies of fetal cardiovascular physiology, which plays a pivotal role in understanding and interpreting fetal CMR studies. Additionally, the authors thoroughly examined the present applications and explored potential future clinical uses (4). Fetal CMR is indispensable for evaluating and addressing fetal cardiac issues, providing valuable insights into fetal cardiovascular functions, and enabling prompt identification and management of congenital heart defects. Its ongoing advancement holds significant potential for improving fetal cardiac care.

In congenital diaphragmatic hernia, fetal Z-Score values were systematically documented (Moon-Grady et al.) Chaoui et al. (5) elucidated recent technological advancements, encompassing the utilization of color Doppler in conjunction with spatiotemporal image correlation (STIC) in the glass-body mode and the matrix probe. These sophisticated techniques precisely visualize cardiac anomalies, particularly within the 4-chamber view and the great arteries. Karmegaraj (6) conducted a comprehensive analytical investigation to ascertain the incremental advantages of 3D/4D STIC fetal echocardiography compared to conventional 2D fetal echocardiography. Particular emphasis was placed on the accuracy of identifying anatomical details critical for surgical decision-making and the prediction of surgical approaches required for fetuses afflicted with double-outlet right ventricles. Amongst contributions in this topic, the Fujian research group methodically synthesized their collective experiences in recognizing anomalies associated with the fetal pulmonary artery, fetal heterotaxy syndrome, and total anomalous pulmonary venous connection (Huang et al., Xue et al., Wu et al.). These evaluations were conducted using 2D echocardiography and color Doppler techniques, with a retrospective analysis encompassing prenatal ultrasonography, neonatal data, and prognostic assessments within a single-center setting. Parallel investigative methodologies were applied to fetal congenital heart disease during the early trimester (Ling et al.).

Kühle et al. provided an exhaustive overview of advanced imaging modalities within the domain of fetal cardiology, with a specific emphasis on evaluating fetal cardiac function. Their discourse comprehensively covered methodologies involving fetal echocardiography and fetal cardiovascular magnetic resonance imaging (MRI). Various techniques were covered, encompassing 2D Echo speckle tracking for strain imaging, 2D phase contrast MRI, 4D flow measurements, ventricular volumetry, and the prospective utility of myocardial strain assessment through fetal MRI. The assessment of hemodynamic parameters, including blood flow dynamics, 3D/4D flow dynamics, and fetal cardiovascular CMR, was underscored for its potential to yield valuable insights into fetal cardiovascular physiology (Udine et al.). While acknowledging the intricacies inherent in fetal CMR gating techniques, the authors accentuated its clinical utility in assessing extracardiac anomalies and fetal cardiovascular hemodynamics. Furthermore, they posited that with the integration of novel technical approaches, fetal CMR holds promise as a more potent clinical diagnostic tool. Zhang et al. presented a comprehensive study involving 130 fetuses afflicted with complete vascular rings. This retrospective analysis leveraged a spectrum of imaging modalities, including eady-State Free Precession (SSFP), SSTSE, real-time SSFP cine, and non-gated PC sequences, for comparative purposes against 260 normal, healthy controls. The investigation provided meticulous methodological insights into characterizing fetal complete cardiovascular rings. Subsequent research endeavours may encompass the execution of large-scale prospective studies intended to establish reference parameters and prognosticate postnatal outcomes, with specific attention to the diverse branching pattern variants. Su et al. contributed a clinical case report delineating an anterior mediastinal teratoma initially detected through fetal echocardiography, subsequently validated by neonatal enhanced CT imaging, and conclusively histopathologically categorized as an immature teratoma (Grade I).

The inception of the cardiovascular profile score pioneered by Huhta (7), marked a seminal development in fetal well-being assessment. Subsequently, the evaluation of fetal cardiac function and the cardiovascular profile has emerged as a critical diagnostic tool, shedding light on conditions such as Ebstein’s anomaly, where it reveals mild to moderate right ventricular (RV) dysfunction, as well as significant clinical manifestations like ascites and scalp edema (Kühle et al.). These assessments play a pivotal role in tailoring precise clinical management strategies. In a pioneering initiative, Crispi’s research group meticulously explored the feasibility and reproducibility of longitudinal strain and strain rate measurements, employing Tissue Doppler Imaging (TDI) and 2D strain techniques. Their extensive work provided a comprehensive overview of the various technologies for assessing fetal heart function through fetal echocardiography (8, 9). A noteworthy study by the Toronto research team underscored the significance of fetal strain analysis, revealing a reduction in combined ventricular output within a preclinical pig model of the artificial placenta (Kühle et al.). In a novel approach, Zhao et al. reported promising results with the fetal HQ technique, particularly in diagnosing fetal ventricular aneurysms and diverticula. Longitudinal parameters, such as annular displacement or velocities, and diastolic parameters, including ductus venosus, E/A ratios, myocardial performance index, or isovolumetric relaxation time, have demonstrated the potential to detect early changes indicative of impaired relaxation and compliance in the fetal heart. This contrasts with changes in ejection fraction and combined ventricular output, which manifest at later stages.

The assessment of fetal cardiac function holds substantial promise in diagnosing, monitoring, and prognosticating various fetal conditions, encompassing primary cardiac diseases and extracardiac anomalies. Speckle Tracking Echocardiography (STE), an advanced echocardiographic imaging modality elucidating tissue motion in the heart via the speckle pattern within the myocardium, has played a pivotal role in these assessments. Li et al. demonstrated the utility of fetal four-chamber global sphericity index, sphericity index, fractional shortening, global longitudinal strain, and fractional area change of the RV, particularly in cases of ductus arteriosus constriction. STIC technology has been introduced over a decade and a half and has substantially advanced the field of fetal echocardiography. Furthermore, recent years have witnessed noteworthy progress in 3D/4D echocardiography, propelled by innovations such as the matrix probe and enhancements in grayscale and color Doppler post-processing. These collective advancements have ushered in improved visualization capabilities and diagnostic precision in ultrasound imaging. Udine et al. performed a mini-review for the state-of-the-art of the fetal CMR. Despite remaining technical barriers, fetal CMR allows anatomic assessment with innovative technologies, including assessment of blood flow, 3D datasets, and 4D flow, providing important insight into fetal cardiovascular physiology.

## 2. Advanced imaging in pediatric heart diseases

Cutting-edge echocardiographic methodologies, including TDI, STE with strain and strain rate imaging, and real-time 3D echocardiography, offer novel insights within pediatric cardiology (10). These innovative approaches can serve as valuable clinical adjuncts, alongside traditional echocardiography, to evaluate myocardial and valvular function in both congenital and acquired heart conditions. CT imaging is frequently employed for vascular evaluation and coronary artery assessment, particularly when CMR may present contraindications, such as metallic implants. Conversely, CMR retains its indispensable role in the field, primarily owing to its proficiency in delivering comprehensive assessments of cardiac anatomy, functional dynamics, and tissue characterization. The concurrent deployment of CT and CMR in pediatric cardiology offers a multifaceted diagnostic paradigm, ensuring optimal patient care and clinical outcomes. 3D printing by CT or CMR extends the capabilities of medical imaging beyond diagnosis and assessment, allowing for the creation of patient-specific models and objects that enhance patient care, medical education, and research. This convergence of technology opens doors to innovative and personalized solutions in healthcare and beyond (11). Ultrafast ultrasound imaging (UUI) is an innovative technique that provides high-speed, real-time visualization of anatomical structures and physiological processes within the body, which has a wide range of clinical applications, including cardiac imaging (for evaluating heart function and blood flow), vascular assessments, musculoskeletal imaging (for examining muscles and tendons during motion), and fetal development studies. While UUI has been primarily used in research settings, it holds great promise for clinical applications, especially in areas where real-time visualization of dynamic processes is crucial for diagnosis and treatment planning (12).

### 2.1. Echocardiographic imaging

Notwithstanding the significant advancements in pediatric echocardiography, it is imperative to underscore the limited number of clinical trials conducted in this domain, many of which have experienced delays in publication. To rectify this situation and foster greater transparency and accessibility of trial findings, a concerted and organized effort is indispensable to promote trial transparency within the pediatric echocardiography community. Rasouli et al. used UUI to quantify local arterial stiffness in a cohort of 20 pediatric cancer survivors undergoing anthracycline treatment, which revealed no discernible influence of anthracycline treatment on local arterial stiffness within the left common carotid artery. These results indicate no observable difference in arterial stiffness between pediatric cancer survivors treated with anthracyclines and individuals in the control group. Mao et al. implemented Z value-based criteria in evaluating left ventricular diastolic dysfunction (LVDD) in pediatric patients afflicted with heart failure or those deemed to be at a heightened risk of heart failure demonstrates promise as a method conducive to the early identification of LVDD. Such an approach holds the potential to facilitate timely therapeutic interventions and medical management. Patent ductus arterious (PDA) constitutes approximately 10–15% of congenital heart diseases. A retrospective investigation involving 222 preterm infants underscores the significant utility of echocardiographic parameters, particularly shunt flow velocities, in acutely predicting the early spontaneous closure of PDA (He et al.).

In the study conducted by Chen et al. an evaluation was conducted to assess left myocardial function alterations subsequent to chemotherapy administered for childhood lymphoma. The primary objective of this investigation was to ascertain the potential utility of speckle-tracking echocardiography in predicting or monitoring cancer treatment-related cardiac dysfunction. The study cohort encompassed 23 children diagnosed with histopathological lymphoma, with age-matched individuals serving as normal controls. Notably, the analysis revealed that global longitudinal strain (GLS) exhibited the highest sensitivity as a predictor for identifying patients at an elevated risk of developing cardiotoxicity associated with anthracycline-based chemotherapy. The echocardiographic assessment unveiled a spectrum of cardiac anomalies, which included pulmonary artery aneurysm, intrapulmonary artery bulge, patent ductus arteriosus, and the concurrent presence of pericardial effusion. Subsequent validation through contrast-enhanced CT affirmed these findings, confirming the presence of a pulmonary artery aneurysm, patent ductus arteriosus, and a minor compression effect on the left main bronchus. In November 2022, surgical intervention was executed, encompassing the reconstruction of the main pulmonary trunk and the repair of the patent ductus arteriosus (Wu et al.). The overarching goal of this review is to advance our comprehensive understanding of Persistent Fifth Aortic Arch (PFAA). This is accomplished by systematically synthesizing key facets, encompassing its embryonic developmental origins, pathological classifications, imaging diagnostic methodologies, and clinical treatment modalities. The fundamental aim is to contribute to refining diagnostic accuracy and treatment approaches of PFAA within the academic and clinical domains (Shan et al.).

Zhou et al. performed a case study of congenital generalized lipodystrophy in an infant girl. The authors combined conventional echocardiography with 2D speckle-tracking to detect infant structural and early functional cardiac changes. Liu et al. used two-dimensional speckle-tracking echocardiography to evaluate the right ventricular longitudinal strain in 58 pediatric patients with pulmonary hypertension cohort. They concluded that right ventricular longitudinal strain is a reliable indicator for evaluating right ventricular functions. Xiang et al. used Z value-based criteria to evaluate LVDD in children with heart failure. They found that the high risk of heart failure may be more conducive to the early identification of LVDD, thereby permitting the possibility of early treatment intervention. Huang et al. reported that an echocardiogram revealed several significant findings, notably, the prolapse of the anterior leaflet, restricted mobility of the septal leaflet, and the presence of severe tricuspid regurgitation, as indicated by a peak gradient pressure of 37 mmHg. It is imperative to underscore that comprehensive preoperative management, when executed effectively and promptly, can be life-saving and potentially significantly enhance the prognosis in such cases.

### 2.2. Multi-modal imaging

Shen et al. conducted a comparative investigation to evaluate coronary artery visibility in a pediatric population. This evaluation utilized a 1.5-T 3D SSFP sequence before and after administering gadolinium-based contrast. The research outcomes revealed that contrast-enhanced imaging significantly improved the detection rate of coronary arteries, particularly in patients below two years. The study underscores the essential role of gadolinium contrast in conjunction with the 3D SSFP sequence for achieving effective coronary imaging in children under two years, with potential benefits extending to the age group 2 to 5 years. Gong et al. compared the diagnosis accuracy of Coarctation of the aorta (CoA) with both transthoracic echocardiography (TTE) and computed tomographic angiography (CTA) in a cohort of 197 cases. They found that CTA is more accurate as a clinical tool for diagnosing CoA; however, TTE with color Doppler can better identify congenital cardiovascular malformations. They suggest combining TTE and CTA would benefit the diagnosis of CoAs. In another study by Yan et al. for 53 CoA cases, the authors used intelligent imaging processing, where measurements of diameter at any part of the aortic 3D models segmented from CTA could be obtained automatically, thus improving the accuracy of imaging analysis and reducing diagnostic subjectivity.

The utilization of computational fluid dynamics (CFD) was employed to examine the alterations in hemodynamics among pediatric patients with CoA. A cohort comprising 99 cases of CoA was categorized into three distinct morphologic patterns, namely Gothic, Crenel, and Romanesque. The study revealed no substantial hemodynamic distinctions observed at the D1/AOA (proximal) and D2/AOA (immediate distal) segments among the three morphological patterns. However, when comparing the three groups, statistically significant hemodynamic differences were evident in the D3/AOA, D4/AOA, and D5/AOA (more distal) segments (Qin et al.).

He et al. used Radiofrequency catheter ablation (RFCA) to treat tachyarrhythmia in children. The authors used “Fluoroscopy integrated 3D mapping,” a new 3D non-fluoroscopic navigation system software to reduce fluoroscopy during the procedure. They quantified the exposure time to fluoroscopy with the new technology, which was significantly lower than conventional RFCA. Alifu et al. performed 3D reconstruction and rendering from CTA DICOM images involving several steps and specialized software, demonstrating that 3D printing technology can produce intricate models of complex intracardiac structures, providing enhanced clarity and precision in anatomical assessment. This advancement is instrumental in facilitating more precise preoperative planning and improving the outcomes of surgical interventions.

## 3. Conclusion

The imperative for a collaborative approach between clinicians and imaging experts in pediatric cardiac care is driven by the need for personalized diagnostic strategies that consider the unique characteristics of each patient. This topic includes studies that carefully assess the strengths and weaknesses of various imaging modalities, minimize risks, and maintain open communication with healthcare teams that can enhance the quality of care and outcomes for pediatric patients with cardiac ailments.
